# Individual Risk Assessment and Prognostication of Outcomes After Corneal Cross-Linking

**DOI:** 10.1155/joph/3678453

**Published:** 2025-07-01

**Authors:** Y. Statsenko, K. Liaonchyk, D. Morozova, R. Voitetskii, M. Pazniak, E. Likhorad, A. Pazniak, P. Beliakouski, D. Abelski, D. Smetanina, G. Simiyu, K. N. V. Gorkom, T. AlMahmoud, H. Aldhaheri, M. Ljubisavljevic

**Affiliations:** ^1^Imaging Platform, ASPIRE Precision Medicine Research Institute Abu Dhabi, Al Ain, UAE; ^2^Radiology Department, College of Medicine and Health Sciences, United Arab Emirates University, Al Ain, UAE; ^3^Neuroscience Platform, ASPIRE Precision Medicine Research Institute Abu Dhabi, Al Ain, UAE; ^4^Microsurgery Department, Eye Microsurgery Centre “Voka”, Minsk, Belarus; ^5^Surgery Department, College of Medicine and Health Sciences, United Arab Emirates University, Al Ain, UAE; ^6^Ophthalmology Department, Tawam Hospital, Al Ain, UAE; ^7^Physiology Department, College of Medicine and Health Sciences, United Arab Emirates University, Al Ain, UAE

**Keywords:** corneal collagen cross-linking, explainable artificial intelligence, intervention outcome, keratoconus, keratometry readings, machine learning, precision medicine, predictive model

## Abstract

**Background and Objective:** Corneal collagen cross-linking (CXL) is a treatment which arrests keratoconus (KC) progression, but its effectiveness differs radically among patients. Herein, we report preoperative diagnostic findings that reflect CXL outcomes and allow physicians to prognosticate treatment efficiency.

**Methods:** In a medical centre, we retrospectively analysed pre- and postoperative data about 107 patients (112 eyes) treated with CXL from January 2018 to December 2022. Exclusion criteria were age below 16 years, a corneal thickness below 400 microns, severe dry eye, other corneal diseases/infections, re-CXL, pregnancy and missing follow-up examinations. All the subjects (79 males and 28 females) were followed for a minimum of 4 and a maximum of 40 months. The study dataset was comprised of 796 cases of clinical assessment, pachymetry, visiometry, refractometry and topography examinations. With these data, we modelled maximum anterior keratometry (*K*_max_) and curvature power of the flat and steep meridians of the corneal anterior surface (*K*_1_ and *K*_2_).

**Results:** Two years after the invasion, corneal curvature coefficients decreased progressively. Then, they remained stable for four months and rose afterwards. In the most accurate *K*_1_, *K*_2_ and *K*_max_ models, the proportion of mean absolute error to the range of values was 1.72, 3.66 and 2.37%, respectively. Pronounced corneal thinning, low best-corrected visual acuity and high *K*_max_ levels predict unfavourable outcomes.

**Conclusions:** The high accuracy of the models advocates for a personalised approach to candidate selection for CXL.

## 1. Introduction

Keratoconus (KC) is an ectatic corneal disorder leading to severely reduced visual acuity and marked impairment in the quality of vision. The incidence of KC varies across different geographic regions [1, 2] depending on diagnostic approaches [3]. Recent studies suggest that KC has become more common due to the reliability of modern diagnostic methods and the increasing public awareness of the disease. The prevalence of KC ranges from 0.17 ÷ 0.2 ^0^/_0000_ in the US and Russia to 4000 ÷ 4790 ^0^/_0000_ in Iran and Saudi Arabia, respectively. The global prevalence is around 138 per 100,000 people [4, 5].

The disease aetiology remains unclear. Recent studies suggest a multifactorial nature of KC resulting from a combination of genetic, biomechanical and environmental factors [6]. KC is high in chromosomal abnormalities and systemic diseases, e.g., Down and Marfan syndromes [2, 7]. The risk of developing KC increases after excessive exposure to ultraviolet radiation and frequent intense eye rubbing caused by allergic reactions or recurrent inflammatory processes [8].

Different pathophysiologic mechanisms account for KC which is a thinning corneal dystrophy. First, degenerative changes take place in keratocytes: these resident cells of the stroma undergo apoptosis and endocytosis [9]. Second, disorganisation of the collagen matrix of the cornea is seen as endoplasmic reticular stress with a widespread decrease in many extracellular matrix proteoglycan core proteins: lumican, keratocan and collagen. The analysis of the KC stromal proteome reveals similarities with neurodegenerative diseases [9]. Third, oxidative stress and chronic inflammation also account for corneal thinning and changes in collagen fibres in KC [9]. Fourth, the pathogenic factors above activate matrix metalloproteinases contributing to further corneal degeneration [10]. The metalloproteinases can destroy the extracellular matrix essential for maintaining corneal structure. In KC, an increased expression of these enzymes and decreased activity of the metalloproteinase inhibitors disrupt the balance necessary for normal remodelling and restoration of corneal tissue [11]. This leads to thinning and weakening of the cornea.

At early stages, the disease may remain asymptomatic. This condition requires timely diagnosis and treatment to prevent progression and deterioration of vision: later, KC manifests with irregular astigmatism, myopia and corneal scarring [12]. Corneal topography is a tool to verify the diagnosis [13, 14]. Treatment is suggested to individuals, taking into account age, sex, disease stage and progression, etc. [4]. The combined analysis of all these data presents a challenge, which worsens disease management and complicates individualised treatment.

With corneal collagen cross-linking (CXL), ophthalmologists can reduce the long-term medical costs of KC treatment by preventing disease progression. In the early stages of KC, CXL minimises the frequency of interventions. In the late stages, it eliminates the need for a more expensive and invasive treatment such as corneal transplantation [15]. A study showed that CXL saves $8677 on treatment and around $35,323 on loss of work capacity. Total savings can range from $150 to $736 million, depending on the prevalence of the disease [16, 17]. According to these data, CXL is a cost-effective solution that improves patients' quality of life [18].

Preoperative keratometry findings, particularly the maximum keratometry (*K*_max_), are the most significant predictors of intervention outcomes. A smaller preoperative *K*_max_ correlates with a higher likelihood of a successful intervention [19, 20]. Additionally, early intervention and a central cone location serve as reliable indicators of long-term results [21]. Preoperative keratometric astigmatism raises the chance of scar formation after CXL [22]. Recent studies have been limited by a lack of comprehensive diagnostic data in models predicting the disease course or treatment efficacy. Future research should encompass a broader range of findings from visiometry, refractometry, pachymetry and keratometry.

In KC, the back surface of the cornea changes earlier than the front surface. As a result, posterior keratometry measurements are more sensitive indicators of disease progression and the effectiveness of CXL [23, 24]. Following the procedure, there is a significant improvement in corneal curvature, corneal astigmatism and other keratometry measurements [25]. Studies using the Pentacam keratometer have reported findings on both anterior and posterior keratometry [26–28]. However, it is important to note that not all keratometers can measure posterior keratometry parameters.

Best-corrected visual acuity (BCVA) is an essential indicator of disease progression both prior to and following treatment [22, 29]. The BCVA results obtained before and after surgery exhibit an inverse relationship [21, 30]. While keratometry is vital for predicting treatment results, examining visiometry, refractometry, pachymetry and topography data can improve the precision of modelling CXL effectiveness.

The objective of this study is to identify a combination of preoperative diagnostic results that adequately reflect the outcomes of CXL. This understanding is essential for creating a risk assessment system, which would be a dependable model trained on the most influential predictors to indicate the most suitable treatment.

To achieve the study's aim, we established and completed the following tasks:

1. Conduct an exploratory analysis of the dataset and evaluate how preoperative keratometry findings influence corneal power after CXL.
2. Investigate the relationship between CXL outcomes and the results of visiometry, refractometry, pachymetry evaluations and topographic data.
3. Develop a model to assess changes in the flat, steep and maximum keratometries following CXL.
4. Identify the most significant predictors of CXL effectiveness in patients with KC.

## 2. Materials and Methods

### 2.1. Study Cohort

In this retrospective study, we reviewed the medical charts of 107 KC patients (112 eyes). A single or both eyes of the patients were affected with KC. The study included only the eyes treated with CXL. The cases of bilateral pathology treated with CXL were recorded in two separate entries in the study dataset. The age of the study participants ranged from 16 to 60, on average 28.62 ± 8.63 years. We observed a disproportion between male and female patients: 79 vs. 28 (74 vs. 26%) aged 28.18 ± 8.18 and 29.75 ± 9.71 years, respectively. The participants underwent CXL at ‘Voka' Medical Centre (Belarus) from January 2018 to December 2022. Each patient was examined one time before the intervention and several times after it, which gave us 796 pairs of cases of pre- and postoperative examinations, in total.

The inclusion criteria were male and female patients of any age, history of KC treated with CXL, availability of results in eye refraction test and completion of ophthalmological examination which included slit-lamp biomicroscopy, pachymetry, keratometry and computerised corneal topography. The exclusion criteria were age below 16 years, a corneal thickness below 400 microns, severe dry eye and other corneal diseases/infections, repeated CXL (re-CXL), pregnancy and missing follow-up examinations. The cases of advanced stages of KC and cicatricial changes in the cornea during biomicroscopic examination were also excluded from the study. Another non-pass criterion was autoimmune diseases in decompensation.

### 2.2. Diagnostic Criteria of KC

In the current study, we applied the following major diagnostic criteria for KC: an increased corneal curvature with a *K*_max_ value of more than 47.0 dioptres (D), a difference between the curvatures of the anterior corneal surface in the two principal meridians of more than 3.0 D (asymmetric astigmatism), thinning of the cornea in the area of its cone-shaped protrusion (less than 490 μm) and a decrease in BCVA worse than 20/20 (1.0).

To diagnose KC, our team paid particular attention to the area where the normally dome-shaped cornea bulges outwards and develops a shape more like a cone. In the corneal topography test, we looked for the topographic patterns characteristic of KC. These include local steepening of the cornea midperipherally below the corneal midline [31], asymmetric bow tie with a skewed radial axis [32], a pear-shaped pull of the central keratoscopy rings with initial steepening of the cornea in the temporal quadrant [33] and irregular astigmatism with inequality of the keratoscopy mires.

Recent studies found keratometry indices to be the most reliable parameters for differentiating between healthy eyes and cases of KC [34]. The features characteristic of KC are central readings of the anterior surface elevation greater than 12 μm and the posterior surface elevation greater than 20 μm.

For borderline diagnosis cases, the following diagnostic findings should raise suspicion for KC: the anterior surface elevation within the range from 6 to 12 μm, the posterior surface elevation in the diapason from 8 to 20 μm, the KC index greater than 1.07 and the central KC index greater than 1.03 [35, 36]. We also applied pachymetry tests to evidence the KC diagnosis. The average pachymetric progression index over 1.6 signalled a definite KC [36]. A cutoff value of 450 μm was used for the minimal pachymetry: corneal thinning below 450 μm indicated the disease. The central keratoconus index (CKI) of more than 1.03 was considered pathological, and the index was a diagnostic criterion for definite KC but not its subclinical form [37, 38].

Although it was not a part of our study, physicians can assess a reliable diagnostic marker of KC: the posterior elevation. The maximal elevation difference of more than 12 μm indicates the subclinical pathology, and the difference over 16 μm is diagnostic for KC [39]. The optimal way for measuring posterior elevation remains a matter of debate. The standard technique takes into analysis the maximum value above the best-fit sphere (BFS) within the central 5 mm of the posterior cornea [40]. To ensure a more accurate assessment, some authors suggest the measurement at the thinnest point of the cornea [41, 42]. Although the posterior elevation can be considered a pretty effective diagnostic method, it cannot be used alone to identify patients with sub- or clinical KC.

### 2.3. Detection of Subclinical KC

The diagnostic criteria for subclinical KC are as follows: a cone-shaped cornea with a slight asymmetry between the eyes, an increased corneal curvature with the *K*_max_ values within the range from 46.5 to 48.5 D, a difference between the curvatures of the anterior corneal surface in the two principal meridians of more than 3.0 D (asymmetric astigmatism) and the thickness of the cornea in the area of its cone-shaped protrusion greater than 490 μm.

From the subclinical and clinical KC criteria, one can see an overlap between the diagnostic diapasons for *K*_max_ values. Regardless of the threshold value adopted, a cutoff-based screening method cannot discriminate between two populations [43]. Multimodal diagnostics is more accurate than unimodal since it relies on a group of measurements rather than an isolated index. To detect KC, ophthalmologists also consider topographic maps, the posterior elevation of the cornea and other parameters.

For the Belin/Ambrosio Display (BAD), the D-value is especially useful for identifying the subclinical KC because it integrates anterior and posterior elevation findings and pachymetric data. The threshold values of 1.7 and 3.0 were used to diagnose the subclinical and clinical forms, respectively [37, 44, 45]. However, some studies revealed that BAD-D values range from 1.6 to 2.6 at the subclinical stage, and they are higher in the full-scale disease [44]. In our study, the thresholds for the ISV index were 37 and 40, correspondingly. We also resorted to the index of vertical asymmetry (IVA). The IVA values of 0.28 and 0.40 helped us to distinguish subclinical and clinical KC. The index of height asymmetry (IHA) greater than 19 was abnormal, i.e., the case was suspicious for the subclinical disease. An IHA over 21 marked the pathology [37]. The index of height decentration (IHD) over 0.014 was abnormal, and an IHD greater than 0.016 was pathological [37].

In the subclinical form, patients may suffer from night blindness due to slight optical irregularities of the cornea. Much like halos, double vision can also happen in early KC. The patients may develop irregular astigmatism, minimal but clinically significant. At the early stage, BCVA remains relatively high (e.g., 20/25 or better). However, patients may still complain that they struggle to see small print in dim light or drive at night. In the subclinical stage, coma and other higher-order aberrations increase, affecting image clarity.

Slit-lamp biomicroscopy was also conducted to diagnose the disease. Early manifestations of KC are multifold. Initially, the disease appears with a ‘fading star' or ‘fireworks' symptom reflecting the rarefaction of keratocytes in the KC matrix at the forming apex. Subsequently, the Kayser–Fleischer symptom occurs due to the accumulation of iron ions forming rings in the paraoptic zone of the cornea [46]. Besides, the iron creates striae of Descemet's membrane—Vogt's striae [47]. With retinoscopy, physicians detect the phenomenon of ‘floating reflexes' or slight irregularity in the early KC. In the late stages of KC, biomicroscopy shows Munson's sign which is a protrusion of the lower eyelid in downgaze [48]. The examination can also reveal a rupture of Descemet's membrane and clouding of the corneal stroma after the acute KC (hydrops cornea).

### 2.4. Methods

#### 2.4.1. Protocol of CXL

We followed the standard Dresden protocol suggested by Wollensak, Spoerl and Seiler [49]. The long-term follow-ups showed that the protocol was safe. The effect of treatment was stable with a low complication rate: corneal opacification and scar formation [50–52], and cases of delayed epithelisation were rare [53, 54].

Recent studies of the Dresden protocol showed a significant decrease in keratometry of the flat and steep corneal meridians and a reduction in maximum keratometry. The latter is considered the most sensitive indicator of disease progression [55–58]. A meta-analysis evidenced the protocol's effectiveness: it stabilises the progression of KC as *K*_max_ decreases, and central visual acuity improves [59, 60]. Other meta-analyses and trials ensured the safety of accelerated corneal cross-linking (ACXL). Still, the standard Dresden protocol had better outcomes in terms of visual acuity and KC stabilisation, particularly in children [61, 62].

According to the standard Dresden cross-linking protocol, we removed the epithelium in the cornea's central zone (7–9 mm) for 30 min. We applied a 0.1% riboflavin solution in 20% dextran to the corneal surface. For the next half an hour, the cornea was exposed to the 365–370 nm ultraviolet with an irradiance of 3 mW/cm^2^, and riboflavin was also reapplied at five-minute intervals. During this time, the cornea absorbed the ultraviolet radiation with an energy density of 5.4 J/cm^2^.

Traditionally, a corneal thickness of less than 400 microns after epithelial removal was considered a contraindication to CXL. Later, hypoosmolar riboflavin solutions came into practice to induce corneal swelling during treatment. The swelling brings thickness to the cornea prior to ultraviolet exposure. The hypotonic riboflavin solution increases the cornea thickness to more than 400 μm if the thickness is less than this after epithelium removal [26]. Due to this, reports on the hypoosmolar riboflavin solutions suggest that an overly thin cornea may no longer be a barrier to treatment [63, 64].

#### 2.4.2. Patient Selection

In the current study, the indication for CXL was the established progression of KC. The progression criteria were as follows: an increase in *K*_max_ by 1.0 D or more over 6–12 months, a rise in the difference between steep and flat keratometry by 1.0 D or more over 1 year, an elevation of average keratometry by 0.75 D or more, a decrease in the central corneal thickness by 2% or more, a drop in spherical equivalent of more than 0.5 D and a reduction in uncorrected visual acuity (UCVA) by one or more lines in the table. The latter criterion is equivalent to the loss of visual acuity that requires new contact lenses more than once per 2 years.

The study included a single case of subclinical stage of KC. The patient was treated with CXL for the following reasons. First, detecting KC at the subclinical stage allows for an early interventional strategy with a favourable long-term prognosis [65]. Second, the current classification of stages is in need of improvement. Recent studies revealed its shortcomings [66]. The classification takes into account only the anterior curvature and apical corneal thickness readings; therefore, it should be updated. The advantages of early treatment and limitations of the clinical staging system can justify CXL in subclinical KC cases.

To grade KC by severity, we adhered to the classification by Amsler as modified by Krumeich [67, 68]. Still, some cases were hard to classify because different parameters could fall into distinct stages. In such cases, we resorted to the ABCD grading system because it transcends the limitations of the Amsler–Krumeich classification which does not consider posterior elevation data and visual acuity. Another weakness of the old classification system is that it relies on apical corneal thickness as opposed to the thinnest point. Besides, it poorly differentiates normal cases from abnormal [69, 70]. The ABCD system and biomicroscopic findings of slit-lamp examination allowed us to overcome these drawbacks (see Table 1).

#### 2.4.3. Measurement Techniques

**Figure 1:**
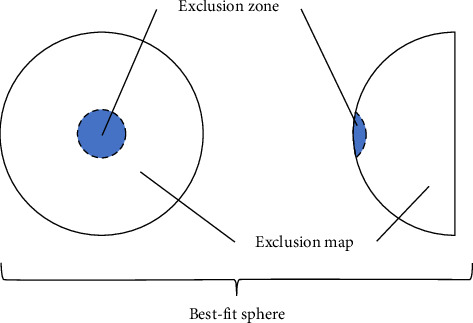
Schematic presentation of exclusion map and zone on corneal surface.

Keratometry data were collected from Marco ARK-1 Series Autorefractor/Keratometer. Topography examination included refractometry indices and elevation back parameters obtained from the corneal apex. The BFS is the most common reference for corneal elevation (see Figure 1). The sphere has an ‘exclusion zone'—a 4.00 mm circle area around the MCT point. The surface area outside the zone is called an ‘exclusion map'. Raw data from the map are used to compute elevation back map (EBM) parameters [71].

To test visual acuity with and without maximum correction in the KC eye, the ophthalmologist or optometrist used Tomey RC-5000 autorefractometer, Tomey TAP-2000 phoropter, trial spherical and cylindrical spectacle lenses of Topcon and a Tomey TCP-1000 LED sign projector. The trial spectacle lenses had positive and negative dioptre equivalents for myopia and hyperopia correction with a step of 0.25 D. Visual acuity was measured according to the size of letters viewed on Golovin–Sivtsev Table with letters and Landolt C characters. The result of the visual acuity test was expressed as a decimal number. A value of 1.0 indicated normal, average vision (100%). The values below and over 1.0 suggested myopia and hyperopia, respectively [72, 73].

### 2.5. Study Methodology

Working on the first task, we analysed associations between pre- and postoperative findings in flat, steep and maximum keratometries (*K*_1_, *K*_2_ and *K*_max_, respectively). Missing values were treated with linear regression imputations. After data preprocessing, we performed an exploratory analysis of the dataset.

All the study subjects were followed for a minimum of 4 and a maximum of 40 months. The observation period was divided into three intervals: less than 6 months, from 6 to 24 months and over 24 months after CXL. To compare the distribution of ophthalmometric findings among the intervals, we applied the Kruskal–Wallis test with the cutoff *p* value of 0.05 (see Table S21 in Supporting Information). The data that did not fall within the range of the 15÷85^*th*^ percentile were considered outliers to be removed from the dataset.

We examined the null hypothesis about the normal distribution of data with the Shapiro–Wilk test. Associations among normally distributed variables were studied with Pearson correlation. For other variables, we used Spearman correlation. The findings weakly associated with *K*_max_ were removed from further analysis.

To tackle the second task, we applied the same approach as in the first task. Specifically, we used the Pearson and Spearman tests to assess relationships of postoperative *K*_1_, *K*_2_ and *K*_max_ values with preoperative keratometry, pachymetry, visiometry, refractometry, and topography data.

To fulfil the third task, we modelled the *K*_1_, *K*_2_ and *K*_max_ values after the intervention with linear and polynomial equations. To determine the model explaining most of the data with a minimum number of parameters, we considered linear, quadratic, cubic and higher degree functions of the time elapsed after CXL. Bayesian information criterion was a method for scoring and selecting the optimal model type.

To complete the fourth task, we trained machine learning (ML) models to prognosticate a postoperative reduction in *K*_1_, *K*_2_ and *K*_max_ from preoperative findings. After feature selection, the study dataset was divided into two parts. The training subset contained 70% of observations, and the remaining cases were used for testing. ML algorithms were trained in a 5-fold cross-validation technique and validated on the training subset. We employed decision tree, random forest, XGBoost, and LightGBM regressors to predict continuous variables. The primary performance metric was the ratio of mean absolute error to the range of values (MAE/ROV). To improve model performance, we optimised hyperparameters with Optuna framework. The framework explores different combinations of hyperparameters to determine the optimal model configuration.

## 3. Results

### 3.1. Association Between Pre- and Postoperative Keratometry

We included patients with subclinical KC (1, 0.76%) and stages 1 to 3-4 KC (see Table S1). A third of the patients had stage 3 KC (41, 31.3%), and almost half of the participants had stages 2 or 2-3 (30, 22.9%, and 27, 20.61%, respectively). The maximum severity in our study was stage 3-4 (13, 9.92%). Patients who came to the follow-ups less than 6 months after CXL had stage 2 or higher. From 6 to 24 months after the procedure, we observed 5 patients (25.00%) with KC stage 1, 8 patients (40.00%) with stages 2 or 2-3 and 7 patients (35.00%) with stage 3. None of the patients with stages 1-2 or 3-4 were examined during this time interval. More than 2 years after the surgery, half of the follow-ups were performed for stage 3 KC (16, 51.61%). 8 patients (25.81%) with stage 2 and 5 patients (16.13%) with stage 2-3 underwent clinical examination beyond the 24-month postoperative period.

**Figure 2:**
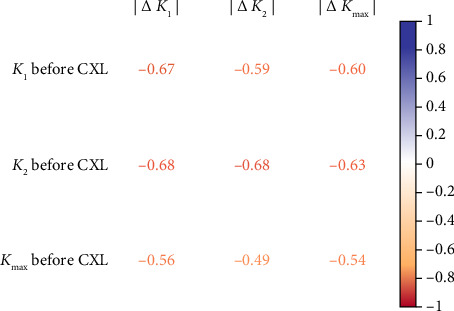
Coefficients of Pearson correlations between postoperative changes in *K*_1_, *K*_2_, *K*_max_ and preoperative values of these parameters (*p* < 0.05).

Preoperative keratometry readings are often used to predict the expected anterior keratometry that follows the treatment. The absolute values of *K*_1_, *K*_2_ and *K*_max_ were negatively correlated with postoperative relative changes (*r* = −0.67, −0.68 and −0.54, respectively), *p* < 0.05 (see Figure 2). The current observation showed multidirectional changes in absolute keratometry values after CXL. In the half-year observation, the cornea's refractive power increased, but then the corneal power reached the preoperative level with nonsignificant changes thereafter (see Table S1).

According to the visiometry test, CXL improved vision: both uncorrected and best-corrected acuity showed a positive trend after the procedure (*p*=0.001 and 0.002, respectively). A drop in UCVA lasted for half a year, and the uncorrected acuity improved later, showing a significant positive trend (*p*=0.001). The same was true for BCVA, whose values initially decreased but later returned to the preoperative level.

The pachymetry test revealed corneal thinning as the structural outcome of CXL. We observed a significant decrease in corneal thickness in the early post-intervention period with a gradual regain in value. The recovery of the structural parameters was slow. In 31 months after the procedure, both CCT and MCT were lower than before the surgery: 459.39 ± 32.60 vs. 479.21 ± 38.35 μm (*p*=0.0445), and 441.13 ± 29.56  vs. 457.74 ± 35.56 μm (*p*=0.0401). The positive dynamics in the structural markers of KC suggests CXL efficiency, which is apparent in low-term observations.

With few exceptions, topograpic and BAD indices did not change pronouncedly after the invasion. The CKI had abnormal values with a downward trend due to the treatment (*p*=0.0328). In the short-term follow-ups, the corneal thickness at the thinnest point (Dt) almost doubled. Later, we observed a continuous decline in the corneal thickness according to BAD-Dt values: 2.83 ± 1.44 vs. 4.23 ± 1.81, 3.86 ± 1.10 and 3.48 ± 1.52 preoperatively and in the short-, middle- and long-term study, respectively (*p*=0.0143).

**Figure 3:**
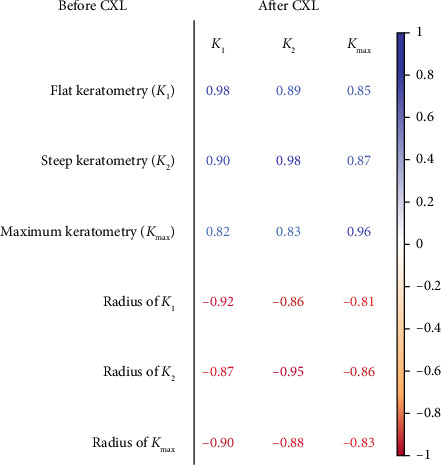
Coefficients of Pearson correlations between flat, steep and maximum anterior keratometry before and after CXL (*p* < 0.05).

The study reported strong associations between pre- and postoperative keratometry findings: r_*K*1_ = 0.98, *p* < 0.05; r_*K*2_ = 0.98, *p* < 0.05 and r_*Kmax*_ = 0.96, *p* < 0.05 (see Figure 3). Patients with smaller preoperative radii of *K*_1_, *K*_2_ and *K*_max_ showed larger postoperative curvature of the cornea (r_*K*1_ = −0.92, *p* < 0.05; r_*K*2_ = −0.95, *p* < 0.05; r_*Kmax*_ = −0.83, *p* < 0.05).

### 3.2. Preoperative Correlates of CXL Effectiveness

**Figure 4:**
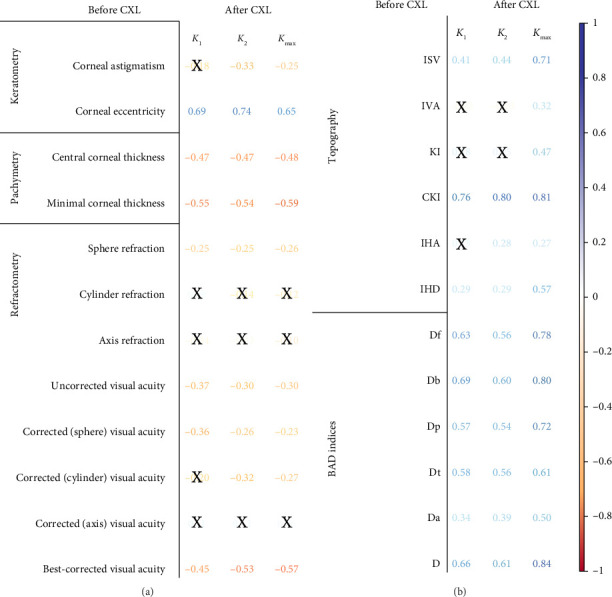
Pearson correlations of postoperative keratometry readings with preoperative keratometry, pachymetry, visiometry, refractometry findings (a), corneal topography and deviation indices (b). Text colour encodes *p* level; weak associations (*p* > 0.05) are crossed out.

We looked for the initial findings markedly associated with postoperative *K*_1_, *K*_2_ and *K*_max_ values (see Figure 4(a)). The values correlated positively with preoperative corneal eccentricity and negatively with other results in visiometry, refractometry, and pachymetry tests, e.g., with sphere refraction (*r* = −0.26 ÷ −0.25; *p* < 0.05). Based on visiometry estimates, the strongest correlate of the flat keratometry findings was pre-CXL BCVA (*r* = −0.45, *p* < 0.05), and the maximal keratometry values got a tighter connection with pre-CXL BCVA (*r* = −0.57, *p* < 0.05). The study revealed a reverse relationship between corneal power and preoperative corneal thickness.

Preoperative topography and BAD deviation indices were also associated with the postoperative keratometry readings (see Figure 4(b)). CKI, BAD-Db and BAD-D positively correlated with *K*_max_ (*r* = 0.81, 0.80 and 0.84, respectively; *p* < 0.05).

**Figure 5:**
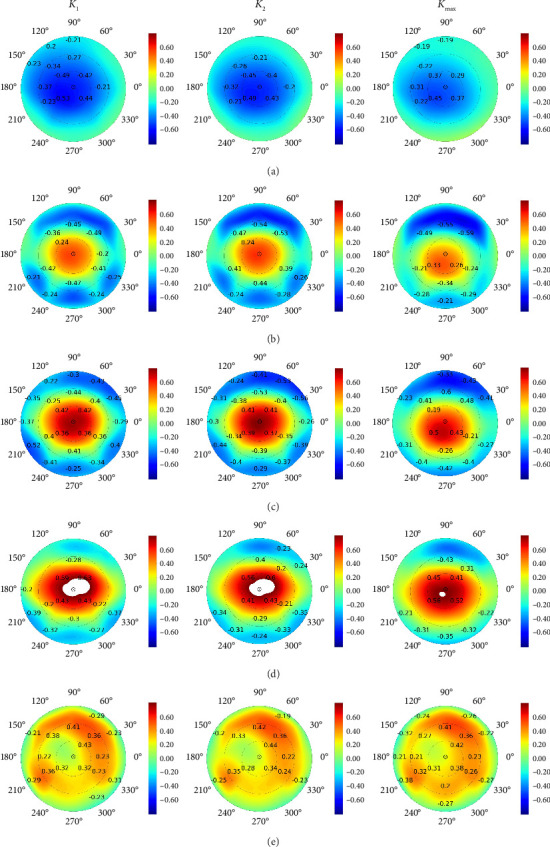
Pearson correlations of postoperative flat, steep and maximum keratometries with preoperative parameters of elevation back maps: corneal thickness (a), posterior elevation (b), posterior elevation with best-fit sphere (c), posterior elevation excluding thinnest zone (d) and excluded thinnest zone of posterior elevation (e). Numbers denote *r*-values of strong correlations (*p* < 0.05); heatmap colour indicates *p* level.

The measurements extracted from EBMs were also tested for similar associations. After the invasion, the flat, steep and maximum keratometries weakly correlated with preoperative corneal thickness (see Figure 5(a)). The postoperative keratometry values were tightly connected to other preoperative data at the EBMs (see Figures 5(b), 5(c) and 5(d)). The associations were more intimate with the map parameters acquired in the corneal centre. However, the ‘excluded thinnest zone of posterior elevation' exhibited an opposite tendency: the peripheral values had a stronger link to *K*_1_, *K*_2_ and *K*_max_ (see Figure 5(e)).

### 3.3. Structural Cornea Changes in Patients With KC After CXL

**Figure 6:**
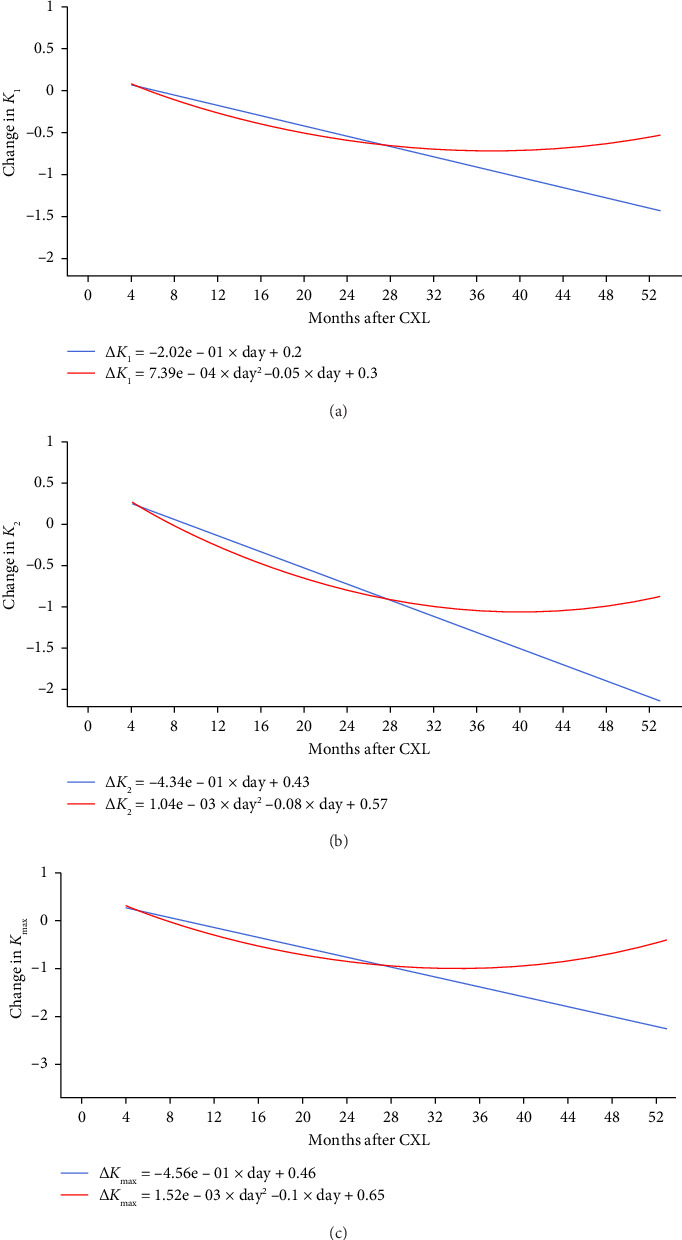
Postoperative changes in flat (a), steep (b) and maximum keratometries (c).

Linear models showed a consistent decrease in *K*_1_, *K*_2_ and *K*_max_ after the invasion (see Figure 6). The slopes were significant at the level of *p* lower than 0.05. Polynomial models described a descending trend in maximum keratometry findings during the first 2 years after CXL. The negative trend was followed by a plateau with a slight subsequent increase in maximum keratometry values thereafter (beyond 28 months after the surgery). A second-degree equation did not boost model performance, and linear model accuracy remained higher with MAE/ROV of 4.05 ± 6.88 vs. 4.1 ± 6.79% (*p*=0.051). This fact may justify a linear dependency between time and change in corneal power after CXL.

### 3.4. Prognostication of CXL Effectiveness in KC Patients

**Figure 7:**
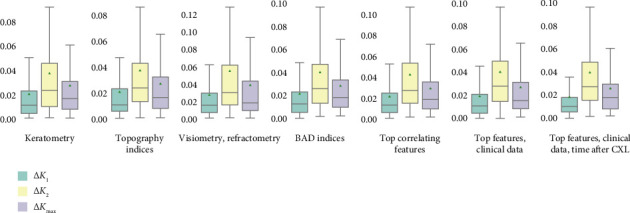
Performance of regression models predicting change in *K*_1_, *K*_2_ and *K*_max_.

Top correlates of postoperative corneal power were considered as its major predictors, and we set the threshold of *r*> 0.3 (*p* < 0.05) for the variables to use in ML. With this threshold, we collected prognostic features from preoperative results in pachymetry, visiometry, refractometry, topography tests and BAD deviation indices. ML models were trained on different sets of data separately and in combination (see Figure 7 and Table S2 in Supporting Information). The most accurate models of *K*_1_ and *K*_max_ were trained on a combination of top correlates and clinicodemographic predictors such as age, sex and time after the invasion (1.72 and 2.37% MAE/ROV, respectively). The most accurate model for *K*_2_ was trained on topographic values (3.66% MAE/ROV). These metrics justify the high reliability of the trained models.

## 4. Discussion

CXL can reduce the rate of KC progression by stabilising collagen fibrils and enhancing the cornea's mechanical strength. The formation of new chemical bonds between the fibril molecules delays the disease progression. In most cases, the advanced stages of KC do not develop after CXL, which emphasises the importance of early intervention to prevent severe complications in the future [74].

CXL considerably lessens the chance of the aggressive KC form commonly treated with corneal transplantation (keratoplasty) [75]. Despite a high graft survival rate (90.4%), keratoplasty possesses limitations and complications [76]. These facts suggest the importance of targeted therapy and treatment choices based on careful risk assessment. Optimal risk management should involve a thorough consideration of the individual risk profile.

### 4.1. Selection of Candidates for CXL

Progressive KC is an indication for CXL. The definition of the progression is as follows: an increase of 1.00 D or more in the steepest keratometry measurement, an increase of 1.00 D or more in the manifest cylinder, an increase of 0.50 D or more in manifest refraction spherical equivalent in one year and reduction of CCT by 5% or more in three consecutive examinations in 6 months [77]. The Global Consensus on Keratoconus and Ectatic Disease recommends CXL in patients experiencing at least two of the following signs: ‘(1) steepening of the anterior corneal surface, (2) steepening of the posterior corneal surface and (3) thinning and/or an increase in the rate of corneal thickness change from the periphery to the thinnest point' [78]. Another indication for CXL is a stable KC intolerant to hard contact lenses [79]. However, the US Food and Drug Authority has not approved CXL for stable cases [80].

The procedure has several contraindications, including pregnancy, epithelial healing disorders, refractive keratotomy, corneal melting disorders, and previous herpes simplex virus keratitis because ultraviolet A may induce herpes reactivation [79]. The treatment is also not recommended for the patients who are likely to have the thinnest point of the cornea below 400 μm after application of riboflavin. Another exclusion criterion for the surgery is keratometry readings exceeding 60.00 D [79]. However, some authors suggest performing CXL on patients with lower keratometry values. For paediatric patients, *K*_max_ steeper than 55 D is already an indication for the procedure [81].

The KC stage can also serve as a criterion for selecting patients who will benefit from CXL the most. The widely used Amsler–Krumeich classification proposes four stages of the disease. Patients will not benefit from CXL if they have grade IV KC with corneal scars [82]. In other disease stages, physicians should perform a thorough risk stratification prior to advising patients to undergo CXL. A recent study showed a more remarkable improvement in BCVA in the early stage of KC than in more severe cases. However, postinterventional topographic indices did not have a pronounced difference across the three stages [83]. Another study reported a significant improvement in flat and mean keratometry among patients with grade I. Among patients with stage III KC, thinnest pachymetry decreased significantly. No pronounced postinterventional changes were observed in cases with stage II and IV [84]. The findings suggest that early initiation of the treatment is more beneficial for progressive cases of KC.

The Amsler–Krumeich classification relies on central anterior curvature and apical thickness measurements that do not reflect subclinical KC cases [70]. Researchers considered the shortcomings of this grading system and developed the Belin ABCD classification which consists of five parameters. Those include the anterior radius of curvature in the 3.0 mm zone centred on the thinnest location of the cornea, the posterior radius of curvature in the 3.0 mm zone centred on the thinnest location of the cornea, thinnest pachymetry in μm and distance BCVA [70]. The sensitivity of this grading system was confirmed in a study that used an ML algorithm to identify subclinical KC. The model trained on the posterior radius of curvature data distinguished between subclinical and clinical KC with ROC AUC = 0.986 [85]. ABCD parameters may also help to identify candidates for CXL: progressive cases have a 7-fold risk of undergoing CXL [86].

### 4.2. Different CXL Protocols

The current study focused on the standard Dresden protocol offering a set of advantages. Numerous clinical studies confirmed its effectiveness in arresting KC progression. Randomised control trials revealed a decrease in *K*_max_ and an improvement in central visual acuity [59, 60]. The positive dynamics in *K*_max_ can be observed 10 years post CXL [87]. Visual acuity continues improving at a 5-year follow-up [88]. Hence, CXL stops KC progression, stabilises the cornea and improves visual functions over a long time. To validate the efficacy of new CXL techniques, long-term studies are required [89].

Studies on ACXL also demonstrated significant clinical improvement: a decrease in *K*_max_ by 1.49 D is a favourable outcome. ACXL outperformed the standard Dresden protocol in cylindrical refraction at a 1-year follow-up. Still, postoperative changes in BCVA are better after the standard intervention [61]. These findings were inconsistent with outcomes of another research in which the standard CXL and ACXL had similar results at one-year follow-ups. Two years after the intervention, the outcomes of CXL were better [62].

The potential drawbacks of CXL are endothelial damage and nerve injury that reduces corneal sensitivity [65]. However, most studies consider it safe for endothelium: the procedure does not change the variation coefficient and the percentage of hexagonal cells. If the treatment adheres fully to the standard Dresden protocol, the exposure of the corneal endothelium to ultraviolet radiation is minimal. In the protocol, riboflavin absorbs ultraviolet A radiation and prevents its damaging effect on endothelial cells [90]. The level of ultraviolet A radiation is also limited to avoid damage to the deep layers. However, other studies claim that cross-linking may damage the corneal endothelium due to severe oxidative stress. It occurs when the cornea is thin, and ultraviolet-A penetrates deeper layers. This situation happens in cases of an improper interventional protocol with a high irradiation dose or pre-existing endothelial dystrophy [91].

In the standard Dresden protocol, the endothelial cell density (ECD) slightly decreases by 0.7%–1.4% 6 months after CXL [92, 93]. The ACXL protocol leads to an ECD reduction due to higher radiation exposure. A study showed a transient decrease in ECD of 6.7% 3 months after ACXL. Still, a complete re-epithelisation occurs in 12 months. The epithelium recovers its standard thickness quicker in patients with post-LASIK ectasia [94]. Hence, the accelerated protocols can also be safe after accurate risk assessment. The CXL procedure poses minimal risk to the corneal endothelium if physicians follow the protocols and conditions for cross-linking [91]. Whatever the protocol, accurate risk assessment requires preoperative diagnostics [95].

### 4.3. Relationship Between Preoperative Ophthalmometric Results and Outcomes of CXL

While corneal thinning may indicate a need for CXL, corneal thickness does not increase in short-term assessments. In the first 2 to 3 years following the procedure, the thickness of the cornea decreases, which does not necessarily reflect the treatment effectiveness. This measurement can only indicate long-term results a decade after CXL [96, 97]. Therefore, we chose *K*_max_ instead of corneal thickness as the key variable for modelling early outcomes using ML.

Our data revealed an inverse relationship between preoperative thickness and postoperative curvature measurements. A recent investigation showed strong correlations between corneal thickness prior to CXL and *K*_2_ following the procedure [98, 99]. Additionally, another study indicated that a significant preoperative *K*_max_ value and a thin cornea are indicators of successful treatment [21, 30].

Visual acuity serves as a functional indicator of KC severity and can also forecast the results of CXL. In the current study, postoperative corneal power showed inverse relationships with initial keratometry, visiometry and refractometry measurements, except for corneal eccentricity. The eccentricity had a positive correlation with *K*_1_, *K*_2_ and *K*_max_. Our findings support the observations made by Chang and Hersh indicating that a lower preoperative BCVA is closely associated with improved *K*_2_ and *K*_max_ values later on [100]. Similar to our results, corneal eccentricity was recently identified as a significant predictor of *K*_max_ following CXL [30].

UCVA is an additional visual function measurement that has not been established as a reliable predictor of CXL results, and there are limited data regarding the relationship between UCVA and *K*_max_. Our research demonstrated that pre-CXL UCVA negatively correlates with postoperative anterior keratometry. Another investigation has also supported UCVA as an indicator of treatment outcomes; however, the significance of these results was low [30]. Based on our findings and those from existing literature, preoperative BCVA appears to be a more robust and commonly accepted marker for assessing the effectiveness of CXL compared to UCVA.

Our findings indicate that postoperative anterior keratometry correlates strongly with BAD indices and weakly with topographic measurements. This association aligns with existing literature suggesting that BAD indices are related to corneal thickness in healthy eyes [101]. The BAD-D value indicates KC progression and can be considered a supplementary criterion for CXL [102]. Other research has demonstrated that topographical indices—such as ISV, IVA and KI improved significantly following successful CXL treatment [96, 103]. Further investigations are needed to enhance understanding of how topographic and BAD indices can predict CXL outcomes.

In KC, the stability of the posterior corneal surface is inferior to that of the anterior surface [26, 104]. Consequently, we sought robust predictors of surgical outcomes based on EBM readings. We examined whether preoperative EBM maps could indicate postoperative changes in anterior keratometry. Our findings revealed a strong correlation between elevation at the corneal centre and postoperative *K*_max_. Other researchers also concur that the effectiveness of CXL is particularly sensitive to the central position of the cone [21, 105].

### 4.4. Determinants of Successful CXL Treatment

#### 4.4.1. Reliability of Preoperative Keratometry Findings in Determining CXL Effectiveness

CXL is less effective in later stages of KC: preoperative *K*_1_, *K*_2_ and *K*_max_ values showed a negative correlation with the magnitude of their reduction following treatment (see Figure 2). Recent research has also indicated significant connections between pre- and postoperative keratometry readings, achieving a significance level of *p*=0.049 [106, 107]. Studies have shown that elevated preoperative *K*_max_ levels suggest a greater risk of KC progression following CXL [20]. Conversely, one study reported an opposite correlation between pre- and postoperative *K*_max_ [108]. In patients with higher *K*_max_ values, short-term outcomes of CXL were more favourable, but the long-term results were poorer [57, 109]. Prior to treatment, increased *K*_2_ values were another crucial indicator of KC progression after CXL [98]. Our analysis further indicates a strong link between the effectiveness of CXL and the preoperative stage of the disease.

Prior research offers limited insights into how CXL impacts the flat, steep and maximum keratometry radii. We observed a significant reverse correlation between these factors before and after the procedure. Additionally, other researchers reported bidirectional changes: the radii reduced at three months and then increased at twelve months following CXL [110]. Future studies need to clarify the changes in the radii of flat, steep and maximum anterior keratometry after CXL.

#### 4.4.2. Other Predictors

Preoperative findings from ophthalmometry and clinical assessments indicate the anticipated results of CXL. We employed top correlates to predict anterior keratometry following CXL and demonstrated a significant predictive value of corneal eccentricity and CKI. Clearly, CXL proves to be more effective for central KC [111]. Based on our analysis, BCVA is another significant factor associated with the success of CXL. Other studies have also indicated a strong negative correlation between preoperative BCVA and the degree of its post treatment improvement [30]. The relationship between anterior keratometry and the BAD-D index is more robust than with other deviation indices [112].

Recent research has not identified a common predictor for the efficiency of CXL, primarily because researchers relied on a limited number of variables. For instance, Badawi and colleagues examined preoperative BCVA, UCVA, *K*_max_, MCT, age and gender as possible indicators of CXL outcomes within univariate frameworks. Their findings highlighted the strong predictive capacity of preoperative BCVA results (*p* < 0.001, B coefficient −0.800 and 95% CI 0.271–0.676) [30]. The diagnostic data consistently correlate with the future disease course. Other researchers predicted KC progression based on *K*_max_ and BAD-D, achieving sensitivities of 49% and 82%, respectively [113]. Kamiya and his team developed high-specific and sensitive deep learning models for disease progression, training them on data from anterior and posterior curvature maps [112].

### 4.5. Delayed Impact of CXL on Anterior Keratometry

Based on our analysis, linear models predicting postoperative changes in *K*_1_, *K*_2_ and *K*_max_ values perform better than polynomial models, although the difference in accuracy is minimal (MAE/ROV: 4.05 ± 6.88 vs. 4.1 ± 6.79%, *p*=0.051). Some researchers contend that polynomial models provide a more precise representation of CXL outcomes [114]. The quadratic function we developed demonstrates a sustained reduction in *K*_max_ for 2 years, followed by a plateau for 4 months, and a reversal of maximum keratometry towards baseline thereafter. According to this model, the effects of CXL diminish after 5 years post-treatment. These patterns align with findings from other research. For instance, 2 years post-treatment, Chatzis and Hafezi reported a marked decrease in *K*_max_; however, the maximum keratometry was not significantly different from the preoperative values 3 years after the surgery [115].

Data regarding long-term outcomes differ across studies. Recent findings indicated a decline in the effectiveness of CXL after 5 years post-treatment [114]. In this analysis, the second-degree linear equation reflects a similar trend (see Figure 6(c)). However, other researchers have demonstrated a sustained reduction in *K*_max_, with this trend lasting up to 10 years [25]. Physicians can anticipate KC progression in individual patients by modelling the long-term effects of CXL from initial assessments. Future research should aim to resolve the discrepancies present in the data.

We developed precise models that predict postoperative maximum keratometry results, achieving a minimal MAE/range of 2.37%. A similar statistical method enabled other researchers to accurately predict *K*_max_ variations over 2-year follow-ups, with R^2^ values of 0.8956 and 0.8382 [116]. These predictive models promote the advancement of personalised medicine by enabling tailored treatment alternatives.

To achieve reputable performance, we developed models utilising a wide range of features linked to CXL outcomes. The regression models incorporated a mix of ophthalmometry readings, clinical information and the duration since CXL. The highest-accuracy models predicted *K*_max_ with a MAE/relative observed variance of 1.72%. Bioengineers should integrate the duration after CXL, ophthalmometry and clinical information to perform a dependable assessment of CXL effectiveness.

### 4.6. Failure of Treatment and Indicators for Re-CXL

Despite overall favourable outcomes of CXL, failure of the procedure happens in 8%–23% of cases at 1-year follow-up [20, 117]. Studies suggest defining post-surgical deterioration as an increase in *K*_max_ of more than 1 D along with possible worsening of BCVA after the first 6 months [118]. Some patients may experience pseudoprogression—temporary worsening due to co-occurring eye diseases and errors in follow-up examinations [119]. Still, physicians can confirm the treatment failure after 6 months of constant worsening of the eye condition after CXL.

Deterioration is associated with various factors, including the type of procedure, corneal thickness, biomechanical properties of the cornea, age and patient compliance [118]. Multiple studies identified high preoperative *K*_max_ (> 57 D) as a risk factor for postoperative progression [20, 120]. Other determinants of progression included female sex, younger age, eye rubbing and thin corneas (< 430 μm) [117, 120, 121]. In addition, the type of CXL may affect the long-term outcome of the intervention: the failure rate is two times higher in patients with ACXL than in individuals with SCXL [20].

To halt postoperative KC progression, patients are advised to undergo re-CXL. The procedure helps avoid keratoplasty associated with complications such as cataract and graft rejection [122]. In several studies, re-CXL was conducted minimum 2 years after the initial treatment [20, 121, 123]. The results were similar to findings on the efficiency of the first CXL: both procedures induced a similar amount of flattening of *K*_max_ [123]. Significant improvement was also exhibited in *K*_1_, *K*_2_ and K_mean_ [121]. Visual acuity, astigmatism and corneal thickness did not change after the repeated treatment [121]. Although re-CXL has positive outcomes, surgeons should consider possible contraindications for the procedure [118].

## 5. Conclusion

• CXL is less effective in the late stages of KC, with a strong negative correlation between preoperative keratometry readings and their absolute change post-intervention (*r* = −0.54, *p* < 0.005 for *K*_max_). Clearly, the outcomes of treatment are enhanced when intervention is performed early.
• Postoperative maximum keratometry positively correlates with preoperative corneal eccentricity, the CKI and BAD-D (*r* = 0.65, 0.81 and 0.84). Conversely, *K*_max_ correlates negatively with preoperative minimal corneal thickness and BCVA (*r* = −0.59 and −0.57). The top correlating features mentioned above have been chosen for ML.
• Linear models for predicting changes in corneal power after surgery are more effective than polynomial models, although their accuracy shows minimal variation (4.05 ± 6.88 vs. 4.1 ± 6.79% MAE/ROV, *p*=0.051). A quadratic function represents a sustained reduction in *K*_max_ for 2 years, followed by a 4-month plateau, and subsequently a reversal of maximum keratometry towards baseline. The effects of CXL diminish beyond 5 years post-treatment.
• These results support a tailored approach for selecting candidates for CXL. Individual risk assessments necessitate a comprehensive evaluation that includes pachymetry, visiometry, refractometry and topography tests. Additionally, physicians should take into account clinical observations and the time elapsed since CXL to reliably predict changes in postoperative *K*_1_ and *K*_max_ using high-accuracy models (1.72 and 2.37% MAE/ROV). The most accurate model for K_2_ was trained on corneal topographic data (3.66% MAE/ROV).

## Figures and Tables

**Table 1:** Classification system used in the current study to grade KC cases.

	Stage 1	Stage 2	Stage 3	Stage 4
*Amsler–Krumeich classification*				
Myopia/astigmatism (D)	< 5	5–8	8–10	Not measurable
*K*_max_ (D)	< 48.0	< 53.0	< 55.0	> 55.0
Posterior corneal curvature (D)	< 59.25	< 65.5	< 68.5	> 68.5
Minimal apical corneal thickness (μm)	> 450	> 400	> 300	< 300

*ABCD grading system*				
Anterior radius of curvature in 3.0 mm zone centred on thinnest location of cornea (mm)	> 7.05	> 6.35	> 6.15	< 6.15
Posterior radius of curvature in 3.0 mm zone centred on thinnest location of cornea (mm)	> 5.70	> 5.15	> 4.95	< 4.95
Thinnest pachymetry (μm)	> 450	> 400	> 300	< 300
Distance best-corrected visual acuity	< 20/20	< 20/40	< 20/100	< 20/400
	(< 1.0)	(< 0.5)	(< 0.2)	(< 0.05)

*Biomicroscopy examination*				
Findings	Cone-shaped cornea	Cone-shaped cornea	Cone-shaped cornea	Cone-shaped cornea
	Vogt's lines	Vogt's lines	Vogt's lines	Vogt's lines
	No opacities	No opacities	No opacities	Central corneal opacities

## Data Availability

The data that support the findings of this study are available from the corresponding author upon reasonable request.

## Nomenclature

Ast: Corneal astigmatism
BAD: Belin/Ambrósio display
ACXL: Accelerated CXL
BCVA: Best-corrected visual acuity
BFS: Best-fit sphere
CCT: Central corneal thickness
CKI: Central keratoconus index
CT: Corneal thickness
CXL: Corneal collagen cross-linking
D: Dioptre
Da: Thinnest point displacement SD
Db: SD of changes in the back elevation
DT: Decision tree
Df: SD of changes in the front elevation
Dp: Pachymetric progression SD
Dt: Thinnest point thickness SD
EBM: Elevation back map
ecc.: Eccentricity of cornea
ECD: Endothelial cell density
IHA: Index of height asymmetry
IHD: Index of height decentration
ISV: Index of surface variance
IVA: Index of vertical asymmetry
*K*_1_: Flat keratometry
*K*_2_: Steep keratometry
*K*_max_: Maximum anterior keratometry
KC: Keratoconus
KI: Keratoconus index
LGBM: Light gradient boosting machine
MCT: Minimal corneal thickness
ML: Machine learning
OCT: Optical coherence tomography
Rf: Radius of *K*_1_
Rm: Radius of *K*_max_
Rper: Average radius of curvature
Rs: Radius of *K*_2_
*R*_min_: Smallest radius of curvature
SD: Standard deviation
UCVA: Uncorrected visual acuity
XGB: Extreme gradient boosting

## Supporting Information

Table S1: characteristics of study participants before and after CXL. Table S2: accuracy of models predicting changes in anterior keratometry after CXL.
