# Supplementary material for: Individual Risk Assessment and Prognostication of Outcomes After Corneal Cross-Linking
Source: J Ophthalmol. 2025 Jul 1;2025:3678453. doi: 10.1155/joph/3678453 (PMC12237562; doi:10.1155/joph/3678453)
Supplement: Supporting Information — Additional supporting information can be found online in the Supporting Information section. [file 3678453.f1.docx]

Supporting Information

Table S1. Characteristics of study participants before and after CXL.

| **Parameter** | **Unit** | **Before CXL** | **After CXL** | | | **p_1-4_** |
| --- | --- | --- | --- | --- | --- | --- |
|  |  |  | **<6 months** | **6-24 months** | **>24 months** |  |
|  |  | **n_1_=131** | **n_2_=50** | **n_3_=20** | **n_4_=31** |  |
| Observation period | month | – | 2.46 ± 1.87 | 12.93 ± 4.76 | 32.3 ± 7.71 | – |
| **Disease stage before CXL:** |  |  |  |  |  |  |
| Subclinical KC | n (%) | 1 (0.8%) | 0 | 0 | 0 | – |
| Stage 1 | n (%) | 15 (11.4%) | 2 (4.0%) | 5 (25.0%) | 1 (3.2%) | – |
| Stage 1-2 | n (%) | 4 (15.0%) | 1 (2.0%) | 0 | 0 | – |
| Stage 2 | n (%) | 30 (22.9%) | 10 (20.0%) | 4 (20.0%) | 8 (25.8%) | – |
| Stage 2-3 | n (%) | 27 (20.6%) | 9 (18.0%) | 4 (20.0%) | 5 (16.1%) | – |
| Stage 3 | n (%) | 41 (31.3%) | 17 (34.0%) | 7 (35.0%) | 16 (51.6%) | – |
| Stage 3-4 | n (%) | 13 (9.9%) | 11 (22.0%) | 0 | 1 (3.2%) | – |
| **KERATOMETRY** | | | | | | |
| Flat keratometry (K1) | D | 45.64 ± 3.83 | 46.95 ± 4.7 | 44.45 ± 2.0 | 45.28 ± 1.92 | 0.0646 |
| Steep keratometry (K2) | D | 49.08 ± 4.54 | 52.02 ± 5.56 | 47.18 ± 2.38 | 48.52 ± 1.2 | **0.0025** |
| Maximum keratometry (Kmax) | D | 56.68 ± 6.44 | 59.75 ± 6.56 | 55.53 ± 3.85 | 56.31 ± 4.5 | **0.0180** |
| Corneal astigmatism | D | -1.79 ± 3.96 | -3.73 ± 4.02 | -2.49 ± 1.75 | -2.13 ± 3.08 | **0.0102** |
| Radius of K1 | mm | 7.45 ± 0.60 | 7.18 ± 0.63 | 7.62 ± 0.46 | 7.47 ± 0.32 | **0.0120** |
| Radius of K2 | mm | 6.90 ± 0.60 | 6.56 ± 0.66 | 7.14 ± 0.48 | 6.96 ± 0.17 | **0.0023** |
| Radius of Kmax | mm | 7.15 ± 0.59 | 6.88 ± 0.65 | 7.34 ± 0.41 | 7.21 ± 0.20 | **0.0187** |
| Corneal eccentricity | – | 0.81 ± 0.41 | 0.95 ± 0.45 | 0.70 ± 0.29 | 0.86 ± 0.28 | **0.0265** |
| Average radius of curvature | mm | 8.01 ± 0.42 | 7.91 ± 0.41 | 8.63 ± 1.38 | 8.09 ± 0.22 | **0.0084** |
| Smallest radius of curvature | mm | 6.02 ± 0.66 | 5.72 ± 0.64 | 6.06 ± 0.57 | 6.03 ± 0.51 | 0.0611 |
| **REFRACTOMETRY** | | | | | | |
| Sphere refraction | D | -3.06 ± 3.93 | -2.12 ± 6.36 | -2.92 ± 2.05 | -3.54 ± 3.23 | 0.5355 |
| Axis refraction | ◦ | 83.65 ± 49.92 | 89.91 ± 59.32 | 79.05 ± 67.58 | 75.07 ± 53.86 | 0.6661 |
| **VISIOMETRY** | | | | | | |
| **Visual acuity:** |  |  |  |  |  |  |
| - Uncorrected | DEC | 0.27 ± 0.23 | 0.19 ± 0.19 | 0.45 ± 0.29 | 0.34 ± 0.27 | **0.0010** |
| - Corrected (sphere) | D | -2.8 ± 3.54 | -4.35 ± 4.11 | -2.85 ± 1.57 | -2.79 ± 2.74 | 0.4197 |
| - Corrected (cylinder) | D | -3.49 ± 2.37 | -4.14 ± 2.87 | -2.10 ± 1.87 | -2.84 ± 2.95 | **0.0024** |
| - Corrected (axis) | ◦ | 90.41 ± 42.63 | 83.48 ± 47.78 | 80.16 ± 44.07 | 97.78 ± 43.66 | 0.2754 |
| - Best corrected | DEC | 0.62 ± 0.25 | 0.51 ± 0.18 | 0.71 ± 0.17 | 0.60 ± 0.22 | **0.0021** |
| **TOPOGRAPHY** | | | | | | |
| ISV | – | 98.15 ± 36.72 | 109.47 ± 30.11 | 94.22 ± 33.67 | 95.1 ± 36.06 | 0.3529 |
| IVA | – | 1.10 ± 0.46 | 1.08 ± 0.49 | 1.16 ± 0.41 | 1.07 ± 0.55 | 0.5732 |
| KI | – | 1.27 ± 0.11 | 1.29 ± 0.11 | 1.24 ± 0.10 | 1.26 ± 0.14 | 0.5374 |
| CKI | – | 1.07 ± 0.06 | 1.09 ± 0.12 | 1.03 ± 0.13 | 1.06 ± 0.03 | **0.0328** |
| IHA | – | 31.22 ± 27.51 | 47.23 ± 43.97 | 38.13 ± 23.71 | 44.97 ± 34.14 | 0.9080 |
| IHD | – | 0.15 ± 0.07 | 0.16 ± 0.06 | 0.15 ± 0.06 | 0.14 ± 0.07 | 0.9474 |
| **BAD INDICES** | | | | | | |
| Df | – | 11.58 ± 6.33 | 13.74 ± 6.93 | 10.61 ± 5.03 | 11.01 ± 6.04 | 0.1528 |
| Db | – | 9.28 ± 5.42 | 10.53 ± 4.86 | 10.89 ± 6.06 | 10.95 ± 6.34 | 0.8853 |
| Dp | – | 9.85 ± 5.04 | 14.06 ± 8.39 | 11.7 ± 3.69 | 12.95 ± 6.50 | 0.8166 |
| Dt | – | 2.83 ± 1.44 | 4.23 ± 1.81 | 3.86 ± 1.10 | 3.48 ± 1.52 | **0.0143** |
| Da | – | 3.26 ± 0.68 | 3.45 ± 0.34 | 3.47 ± 0.22 | 3.32 ± 0.48 | 0.8230 |
| D | – | 9.50 ± 3.45 | 11.1 ± 3.52 | 9.42 ± 2.59 | 10.01 ± 3.60 | 0.2620 |
| **PACHYMETRY** | | | | | | |
| **Corneal thickness:** |  |  |  |  |  |  |
| - central | µm | 479.21 ± 38.35 | 448.28 ± 42.18 | 452.41 ± 30.33 | 459.39 ± 32.60 | **0.0445** |
| - minimal | µm | 457.74 ± 35.56 | 428.20 ± 39.86 | 439.47 ± 28.31 | 441.13 ± 29.56 | **0.0401** |

Table S2. Accuracy of models predicting changes in anterior keratometry after CXL.

| **Modality** | **Predictors** | **Target** | **Model architecture** | | | | **Mean±SD** |
| --- | --- | --- | --- | --- | --- | --- | --- |
|  |  |  | **LGBM** | **Random Forest** | **Decision Tree** | **XGB** |  |
| **Keratometry,**  **pachymetry** | Ecc. of the cornea, | K1 | 2.20±4.82 | 2.02±4.81 | 2.36±5.07 | 2.18±4.69 | 2.2±4.8 |
|  | CCT, MCT | K2 | 3.83±4.25 | 3.73±4.54 | 3.94±4.49 | 4.03±5.35 | 3.9±4.3 |
|  |  | Kmax | 2.85±3.70 | 2.65±3.74 | 3.15±3.87 | 2.88±3.75 | 2.9±3.6 |
| **Visiometry, refractometry** | UCVA, BCVA, corrected visual acuity, sphere and cylinder refraction | K1 | 2.81±5.6 | 2.78±5.71 | 2.93±5.56 | 3.12±6.24 | 2.9±5.7 |
|  |  | K2 | 5.76±8.73 | 5.42±8.66 | 5.75±8.73 | 5.78±8.48 | 5.7±8.4 |
|  |  | Kmax | 4.06±7.46 | 3.85±7.44 | 4.12±7.39 | 4.08±7.32 | 4.0±7.3 |
| **Topography indices** | ISV, KI, CKI, IHD | K1 | 2.25±4.85 | 2.04±4.87 | 2.31±4.77 | 2.22±4.90 | 2.2±4.8 |
|  |  | K2 | 4.01±4.60 | 3.76±4.59 | 4.01±4.44 | 3.66±4.72 | 3.9±4.4 |
|  |  | Kmax | 2.84±3.78 | 2.74±3.77 | 2.96±3.56 | 2.74±3.60 | 2.8±3.6 |
| **BAD indices** | Df, Db, Dp, Dt, Da, D | K1 | 2.22±4.82 | 2.1±4.76 | 2.29±4.72 | 2.28±4.83 | 2.2±4.7 |
|  |  | K2 | 4.22±4.80 | 3.98±4.87 | 4.17±4.34 | 4.08±5.66 | 4.1±4.7 |
|  |  | Kmax | 2.86±3.71 | 2.78±3.88 | 3.20±3.72 | 2.74±3.62 | 2.9±3.6 |
| **Top correlating features** | Ecc. of the cornea, | K1 | 2.26±4.77 | 2.1±4.79 | 2.25±4.82 | 2.40±4.96 | 2.3±4.8 |
|  | BCVA, CKI, BAD-D | K2 | 4.47±4.68 | 4.20±4.81 | 4.24±4.63 | 4.51±5.68 | 4.4±4.5 |
|  |  | Kmax | 2.90±3.55 | 2.82±3.68 | 3.26±3.83 | 3.08±3.99 | 3.0±3.6 |
|  |  |  |  |  |  |  |  |
| **Top features +**  **clinical data** | Sex, age on CXL day,  bilateral pathology, stage, disease duration | K1 | 2.05±4.71 | 1.93±4.73 | 2.07±4.83 | 2.21±4.78 | 2.1±4.7 |
|  |  | K2 | 4.06±4.48 | 3.91±4.70 | 4.52±4.67 | 4.19±6.04 | 4.2±4.5 |
|  |  | Kmax | 2.95±3.86 | 2.59±3.64 | 2.86±3.76 | 2.99±4.06 | 2.8±3.6 |
| **Top features +**  **clinical data +**  **time after CXL** | Sex, age on CXL day,  bilateral pathology, stage, disease duration | K1 | 1.78±4.54 | 1.72±4.59 | 2.32±4.83 | 1.88±4.72 | 1.9±4.6 |
|  |  | K2 | 4.04±4.23 | 3.81±4.28 | 4.34±4.83 | 3.86±5.32 | 4.0±4.0 |
|  |  | Kmax | 2.61±3.28 | 2.55±3.45 | 3.10±3.81 | 2.37±3.34 | 2.7±3.2 |
